# Enhancing Undergraduate Ophthalmology Teaching Through Virtual Reality: Integrating the Eyesi Direct Ophthalmoscopy Simulator

**DOI:** 10.7759/cureus.100021

**Published:** 2025-12-24

**Authors:** Kraig Jamieson, Eilidh Edmiston

**Affiliations:** 1 Ophthalmology, NHS Tayside, Dundee, GBR; 2 General Surgery, NHS Tayside, Dundee, GBR

**Keywords:** career motivation, educational technology, immersive learning, ophthalmology, ophthalmology teaching, simulation-based education, simulation in medical education, undergraduate medical students, virtual reality in medical education, virtual reality simulation

## Abstract

Introduction: Ophthalmoscopy is a key component of both neurological and general medical examinations, yet medical students consistently report low confidence and competence in performing the skill. Newly qualified doctors are expected to perform direct ophthalmoscopy with indirect supervision, yet opportunities for students to develop this skill are often limited due to reduced ophthalmology teaching and limited access to suitable patients. Simulation-based education has emerged as a valuable adjunct to clinical teaching, providing opportunities for deliberate practice, feedback, and safe skill acquisition. This study evaluated the educational impact of the Eyesi Direct Ophthalmoscopy simulator (Haag-Streit Group, Köniz, Switzerland) on medical students' confidence and perceived competence in fundoscopy.

Methods: 122 undergraduate medical students from the University of Dundee (fourth-year medical students and third-year ScotGEM students) attended a two-hour ophthalmology workshop in groups of four to eight. Each session incorporated a short teaching component followed by hands-on practice using the Eyesi simulator. Students examined simulated patients with normal fundi and various pathological findings, including swollen optic discs, central retinal vein occlusion (CRVO), central retinal artery occlusion (CRAO), and proliferative diabetic retinopathy (PDR). Immediate feedback was provided by facilitators, allowing for guided, deliberate practice. After the sessions, participants were invited to complete an anonymous online questionnaire assessing perceived confidence, learning effectiveness, and engagement, accessed via QR code or email link. Participation was voluntary, and feedback was collected anonymously.

Results: All 122 students provided feedback. Feedback indicated that 98% of students felt confident performing ophthalmoscopy under supervision after training. One hundred percent reported increased competence in fundoscopy, and 100% recommended integration of simulator training into the undergraduate curriculum. Qualitative feedback highlighted realism, repeated practice without patient discomfort, and the value of immediate visual feedback from facilitators who could observe the same fundus view.

Conclusion: The Eyesi simulator offers an effective and engaging method for teaching ophthalmic examination skills to undergraduate medical students. By combining realistic visualisation, varied pathology exposure, and immediate feedback, the simulator fosters confidence and diagnostic competence while reducing the anxiety often associated with clinical fundoscopy. Integrating simulator-based teaching into undergraduate curricula aligns with General Medical Council (GMC) outcomes and addresses key educational challenges in ophthalmology. The results support wider adoption of virtual reality simulation as a core component of undergraduate ophthalmology training.

## Introduction

Direct ophthalmoscopy remains an essential clinical skill for identifying optic nerve head, retinal, and vascular pathology. It is a critical component of neurological, cardiovascular, and general medical examinations, allowing clinicians to assess conditions such as swollen optic discs, hypertensive retinopathy, and diabetic eye disease. According to the General Medical Council (GMC) Outcomes for Graduates, newly qualified doctors must be able to perform direct ophthalmoscopy under indirect supervision [1]. Despite this requirement, numerous studies have shown that medical students and junior doctors frequently report low confidence and limited competence in performing the procedure [2,3].

Several factors contribute to this training gap. Ophthalmology often receives minimal curriculum time in undergraduate medical education, typically limited to short rotations or lecture-based modules [4,5]. Opportunities for direct patient examination are constrained by clinical pressures, infection control measures, and patient discomfort during novice examinations [3,6]. Direct ophthalmoscopy also has a steep learning curve, with technical challenges including correct alignment, focusing, and interpretation of findings [6,7]. Consequently, many students graduate without sufficient exposure or confidence to perform fundoscopy effectively in clinical settings [2].

Simulation-based education has emerged as an important tool to address these challenges. By providing a controlled, reproducible, and psychologically safe environment, simulation enables deliberate practice and immediate feedback, both essential for procedural skill acquisition [8]. Previous work has demonstrated that simulation not only enhances technical proficiency but also promotes learner engagement and self-efficacy [9]. Grounded in Kolb's experiential learning theory, simulation supports the full learning cycle of active experimentation, reflective observation, abstract conceptualisation, and practical application [10]. This model is particularly valuable for visuomotor skills such as ophthalmoscopy, where confidence and hand-eye coordination develop through repetition. Within ophthalmology, simulation has evolved from static manikins and photographic models to advanced technologies capable of reproducing real-world optics and pathology, with studies reporting improvements in diagnostic accuracy, pattern recognition, and procedural confidence among learners [7,11]. The Eyesi Direct Ophthalmoscopy simulator (Haag-Streit Group, Köniz, Switzerland) is one of the most widely adopted high-fidelity tools for this purpose.

The Eyesi Direct Ophthalmoscopy simulator provides a computer-based platform that replicates the optical properties, examination technique, and diagnostic challenges of fundoscopy through a binocular model head with adjustable pupil size and refractive settings connected to a digital display generating immersive, three-dimensional retinal environments [9,11]. Learners can examine a wide range of normal and pathological fundi and progress through structured modules that develop technical skills such as alignment, focusing, and systematic navigation while receiving objective, real-time feedback. Integrated performance-tracking metrics, including examination completeness, steadiness, light exposure, and accuracy of pathology identification, enable deliberate practice, longitudinal skills development, and standardised assessment [9]. Facilitators can view the learner's visual field in real time, supporting targeted coaching and reducing the cognitive opacity inherent in traditional ophthalmoscopy teaching [11]. Collectively, these features position the Eyesi as a powerful tool for bridging the gap between theoretical understanding and practical competence in ophthalmoscopy training.

The University of Dundee introduced the Eyesi simulator to support undergraduate ophthalmology teaching more than five years ago. Although the technology was available within the institution, it had not been routinely integrated into structured teaching for all students. Recognising this gap, supplementary simulation-based workshops were developed to enhance clinical skill development and student confidence.

This study aimed to evaluate the educational value of these sessions by exploring students' perceptions of the Eyesi simulator's impact on learning, confidence, and engagement in ophthalmology. Specifically, it sought to determine whether simulation could help bridge the gap between theoretical knowledge and practical competence in fundoscopy.

## Materials and methods

This descriptive, cross-sectional study evaluated undergraduate medical students' experiences of simulation-based ophthalmoscopy teaching delivered using the Eyesi Direct Ophthalmoscopy simulator. A total of 122 students from the fourth-year MBChB programme and the third-year ScotGEM cohort at the University of Dundee participated. Teaching occurred within the ophthalmology department as part of the students' scheduled ophthalmology placements. Sessions were delivered in small groups of four to eight students within departmental teaching areas, and although the workshops formed part of the placement timetable, completion of feedback was voluntary and had no influence on academic progression.

Each two-hour session began with a brief recap of core ophthalmology principles and a short introduction to the Eyesi simulator to familiarise students with the equipment, followed by a concise overview of the direct ophthalmoscopy technique and key fundoscopic signs. The main component of the workshop consisted of supervised, hands-on use of the Eyesi system, with students examining simulated cases including normal fundi, swollen optic discs, central retinal artery occlusion, and proliferative diabetic retinopathy. Facilitators experienced in ophthalmology teaching provided real-time coaching on examination technique and interpretation, encouraged students to verbalise their observations, and supported reflective discussion. Each workshop concluded with a short debrief to consolidate key learning points and address common difficulties.

To evaluate the educational impact of these sessions, students were invited to complete an anonymous questionnaire at the end of each session, administered via Microsoft Forms and accessible by either QR code or emailed link. The questionnaire was developed by the authors specifically for this study. It included a multiple-choice question asking whether students felt able to perform ophthalmoscopy under indirect supervision, followed by a series of statements rated on a five-point Likert scale ranging from "strongly disagree" to "strongly agree". These statements assessed enjoyment of the session, understanding of the teaching aims, confidence in performing direct ophthalmoscopy, confidence in using a slit lamp, perceived increase in ophthalmology knowledge, interest in ophthalmology as a specialty, and whether facilitators had adequately addressed questions. An additional Likert-scale item asked whether the Eyesi simulator should be incorporated into the core integrated teaching programme. The questionnaire concluded with open-ended questions exploring the most enjoyable aspects of the session, suggested improvements, and any additional comments. No demographic, identifiable, or academic performance data were collected. The questionnaire used in this study was entirely self-designed for the purposes of this project and was not adapted from any previously published or licensed instrument, and all items, those employing a traditional Likert response format [12], are free to use and do not require permissions or licensing. The full questionnaire is provided (Table 1).

**Table 1:** Post-simulation questionnaire assessing student perceptions of the Eyesi Direct Ophthalmoscopy simulator This table presents the complete self-designed questionnaire used to evaluate students' perceptions following the Eyesi Direct Ophthalmoscopy simulation session. The questionnaire was created specifically for this study and is original, free to use, and not derived from or dependent on any licensed or proprietary assessment tools. The questionnaire includes the following: (1) a multiple-choice item asking whether students felt able to perform ophthalmoscopy under indirect supervision ("yes", "no", or "not sure"); (2) six five-point Likert-scale statements assessing enjoyment of the teaching session, understanding of session aims, confidence in performing ophthalmoscopy, perceived improvement in ophthalmology knowledge, and interest in further ophthalmology training, with response options ranging from "strongly disagree" to "strongly agree" (using a standard Likert response format [12]); (3) a multiple-choice question regarding interest in attending additional ophthalmology simulation sessions ("yes" or "no"); and (4-6) three open-ended free-text questions inviting students to describe the most enjoyable aspect of the session, suggest improvements, and provide any additional feedback. As a self-developed instrument, this questionnaire has not been previously validated.

Question number	Question	Options
1	Do you feel able to perform ophthalmoscopy with indirect supervision?	Yes/No/Not sure
2a	I enjoyed this teaching session	Strongly disagree/Somewhat disagree/ Neutral/Somewhat agree/Strongly agree
2b	I understand the aims of this teaching session	Strongly disagree/Somewhat disagree/ Neutral/Somewhat agree/Strongly agree
2c	I feel more confident performing an ophthalmoscopy exam	Strongly disagree/Somewhat disagree/ Neutral/Somewhat agree/Strongly agree
2d	I feel this session has increased my ophthalmology knowledge	Strongly disagree/Somewhat disagree/ Neutral/Somewhat agree/Strongly agree
2e	This session has increased my interest in ophthalmology as a specialty	Strongly disagree/Somewhat disagree/ Neutral/Somewhat agree/Strongly agree
2f	I believe simulator training should be implemented as part of standard undergraduate ophthalmology teaching	Strongly disagree/Somewhat disagree/ Neutral/Somewhat agree/Strongly agree
3	Would you be interested in attending additional ophthalmology sessions if these were offered to you?	Yes/No
4	What was the most enjoyable part of the session?	[free text]
5	Is there anything that can be improved in the session?	[free text]
6	Is there any other feedback you would like to provide?	[free text]

In line with local institutional guidance, this project was classified as a service evaluation of an anonymised educational activity that did not alter the established curriculum; therefore, formal ethical approval was not required. Participation was voluntary, responses were fully anonymous, and no identifiable or assessment-related data were collected.

## Results

One hundred and twenty-two medical students completed the anonymous questionnaire. Ninety-eight percent (120/122) of the students felt able to perform ophthalmoscopy with indirect supervision after the simulation session. Additionally, 89% (108/122) of the students strongly agreed that they felt more confident performing fundoscopy after simulator training, with the remaining 11% (14/122) somewhat agreeing, indicating that all participants reported benefit from the sessions. Similarly, 87% (106/122) of the students strongly agreed that the simulator training had increased their clinical knowledge in ophthalmology, while the remaining 16 students somewhat agreed.

Moreover, 79% of the students expressed interest in attending additional sessions, having experienced immediate benefits from hands-on practice. Notably, 100% of the students who were asked the statement "I believe simulator training should be implemented as part of standard undergraduate ophthalmology teaching" responded with "strongly agree" or "agree". Participant responses to the questionnaire are summarised in Table 2.

**Table 2:** Student-reported confidence, knowledge, and engagement following the Eyesi Direct Ophthalmoscopy simulation (n=122) Responses from medical students following the Eyesi Direct Ophthalmoscopy simulation. "Positive responses" include both "strongly agree" and "somewhat agree". Percentages indicate the proportion of students reporting a positive response for each questionnaire item, demonstrating generally favourable perceptions of the simulation and support for its integration into undergraduate ophthalmology teaching.

Questionnaire item: "After this session..."	Strongly agree	Somewhat agree	Total positive responses	Percentage of positive responses
I feel able to perform ophthalmoscopy under indirect supervision	120	-	120	98%
I feel more confident performing fundoscopy	108	14	122	100%
I can confirm I enjoyed this session	103	19	122	100%
I believe my clinical knowledge in ophthalmology has increased	106	16	122	100%
I am more interested in ophthalmology as a specialty	31	46	80	66%

Qualitative feedback highlighted the value of simulation for visualising disease processes, practising repeatedly without patient discomfort, and developing hand-eye coordination. Students particularly appreciated the realism of the simulator and the opportunity for immediate feedback. Several participants suggested integrating simulator-based learning earlier in the medical curriculum and providing more frequent opportunities for practice.

## Discussion

This study supports the role of simulation-based learning in enhancing ophthalmology education for undergraduate medical students. Feedback indicates that the Eyesi simulator increases confidence and perceived competence in direct ophthalmoscopy, aligning with prior evidence that high-fidelity simulations facilitate effective learning through deliberate practice, structured feedback, and repeated exposure to clinical scenarios [2,8].

Kolb's experiential learning framework underpins this approach, allowing learners to progress through active experimentation, reflective observation, abstract conceptualisation, and practical application [10]. In this study, the combination of hands-on practice, immediate facilitator feedback, and reflective discussion enabled students to consolidate both technical skills and diagnostic reasoning.

Consistent with previous ophthalmology education research, the Eyesi simulator provided a realistic, immersive platform for skill development, enhancing both engagement and understanding of fundoscopic pathology [5,7]. Students valued the opportunity to practise safely and repeatedly, which aligns with Biggs and Tang's principles of constructive alignment, ensuring that learning activities, assessment, and intended outcomes are closely matched [6]. The small-group, near-peer teaching format further encouraged active participation and peer learning, echoing evidence that peer-led education fosters motivation, retention, and confidence in clinical skill acquisition [11,13-16].

An important benefit of simulation is the reduction in learner anxiety associated with performing procedures on real patients [8]. By providing a psychologically safe environment, students can focus on technique, visual recognition of pathology, and diagnostic reasoning without concern for patient discomfort [7]. Nevertheless, simulation should complement rather than replace traditional patient-based learning. Direct patient contact remains essential for developing communication skills, professional behaviours, and the nuanced aspects of clinical examination that cannot be fully replicated virtually. In this study, simulation was used as an adjunct to conventional patient-based teaching rather than a replacement, allowing students to practice fundoscopy safely while still engaging in real patient examinations.

The improvements in confidence and perceived preparedness observed in our cohort mirror trends reported in the wider literature. Simulation-based ophthalmology teaching offers a safe, reproducible environment for early skill acquisition, and recent studies have demonstrated measurable benefits for both competence and learner self-efficacy. A recent study has shown that structured simulation training significantly enhances medical students' confidence and examination performance in fundoscopy, supporting the educational value of approaches such as the Eyesi Direct Ophthalmoscopy simulator used in this study [16]. Our data similarly suggest that simulation may help bridge the gap between theoretical teaching and limited opportunities for direct ophthalmoscopy in clinical settings.

Limitations of this study include its reliance on subjective, self-reported outcomes and the absence of an objective assessment of skill improvement. As this project was designed as a service evaluation focusing on student perceptions following the implementation of the Eyesi simulator, no pre-simulation or baseline assessment was undertaken. Consequently, outcomes reflect perceived confidence and competence rather than objectively measured skill acquisition. Future research could incorporate pre- and post-simulation competency assessments, longitudinal follow-up to evaluate skill retention, and comparisons between simulator-trained and traditionally trained cohorts. Additionally, multi-centre studies could explore generalisability across different curricula and student populations.

Overall, these findings support the integration of simulation-based ophthalmoscopy training within undergraduate curricula. By combining immersive virtual reality technology, structured feedback, and small-group facilitation, simulation addresses key educational gaps in ophthalmology teaching and prepares students to perform fundoscopy confidently and competently in clinical practice. While simulation has been more widely applied in postgraduate education, emerging evidence also supports its value for undergraduate ophthalmology teaching [7,9,11,16].

## Conclusions

The Eyesi Direct Ophthalmoscopy simulator was well received by undergraduate medical students and significantly enhanced their confidence, engagement, and perceived competence in performing fundoscopy. Simulation-based teaching offers a safe, repeatable, and effective adjunct to traditional patient-based learning, particularly for technically challenging skills that require substantial practice. Integration of virtual reality simulation into undergraduate ophthalmology curricula has the potential to improve preparedness for clinical practice, foster early skill acquisition, and align with GMC competencies for newly qualified doctors. Future studies incorporating the objective assessment of skill acquisition and long-term retention could further define the role of simulation in medical education.
